# Clinical Factors Associated With Chronic Pain in Communicative Adults With Cerebral Palsy: A Cross-Sectional Study

**DOI:** 10.3389/fpain.2020.553026

**Published:** 2020-11-24

**Authors:** Eric M. Chin, Colleen Lenz, Xiaobu Ye, Claudia M. Campbell, Elaine Stashinko, Lauren L. Jantzie, Gwendolyn Gerner, Alexander H. Hoon, Shenandoah Robinson

**Affiliations:** ^1^Department of Neurology and Developmental Medicine, Kennedy Krieger Institute, Baltimore, MD, United States; ^2^Department of Pediatrics, Johns Hopkins University School of Medicine, Baltimore, MD, United States; ^3^Phelps Center for Cerebral Palsy and Neurodevelopmental Medicine, Department of Neurology and Developmental Medicine, Kennedy Krieger Institute, Baltimore, MD, United States; ^4^Department of Neurosurgery, Johns Hopkins University School of Medicine, Baltimore, MD, United States; ^5^Department of Psychiatry and Behavioral Sciences, Johns Hopkins Bayview Medical Center, Baltimore, MD, United States; ^6^Department of Neurology, Johns Hopkins University School of Medicine, Baltimore, MD, United States; ^7^Department of Neuropsychology, Kennedy Krieger Institute, Baltimore, MD, United States

**Keywords:** cerebral palsy, chronic pain mechanisms, sensorimotor dysfunction, adults with cerebral palsy, cerebral palsy and pain

## Abstract

Chronic pain is prevalent in adults with cerebral palsy. We aimed to explore associations between chronic pain and somatosensory, motor, cognitive, etiologic, and environmental factors in adults with cerebral palsy. This cross-sectional study enrolled 17 adult participants with cerebral palsy (mean age 31 years; 8 female; Gross Motor Functional Classification Status levels I-V) able to self-report and 10 neurotypical adult volunteers (mean age 34 years; 9 female). Participants reported pain characteristics, demographics, and affective factors. Physical examination included somatosensory and motor evaluation. Between-group comparisons used a ranksum test, and correlation analyses estimated effect size in terms of shared variance (ρ^2^). Individuals with cerebral palsy reported greater pain intensity, neuropathic qualities, and nociceptive qualities than control participants. Higher pain intensity was associated with female gender (ρ^2^ = 16%), anxiety/depression symptoms (ρ^2^ = 10%), and lower household income (ρ^2^ = 19%). It was also associated with better communicative ability (ρ^2^ = 21%), spinothalamic (sharp/temperature) sensory abnormalities (ρ^2^ = 33%), and a greater degree of prematurity (ρ^2^ = 17%). This study highlights similarity of chronic pain associations in people with cerebral palsy with patterns seen in other populations with chronic pain. Spinothalamic sensory abnormalities suggest central pain mechanisms.

## Introduction

Chronic pain is reported in 2/3 of adults with cerebral palsy (CP) and is recognized as a major factor impacting quality of life [1]. Mechanisms are poorly understood—limiting the development of effective, evidence-based treatments [2].

While motor impairment is a defining characteristic of CP, there is converging evidence that the underlying etiology of chronic pain extends beyond musculoskeletal pathology. Musculoskeletal pain would be expected to elicit nociceptive pain descriptors (“sore,” “achy,” or “tender”), yet a substantial subset of individuals with CP additionally report neuropathic pain qualities (“sharp,” “burning,” or “stabbing”) [3]. Supporting the concept of neuropathic pain, somatosensory abnormalities are prevalent in individuals with CP. Quantitative sensory testing (QST) in children with CP often shows mechanical and thermal hypoaesthesia as well as mechanical hyperalgesia [4]. The pattern of sensory abnormalities may shift over the lifespan [5].

Additional somatosensory abnormalities in people with CP extend beyond those which are typically examined in pain research, including deficits in proprioception, stereognosis, and tactile discrimination [5]. However, the relationship between individual somatosensory characteristics and pain phenotypes is not clear. It has been suggested that sensory abnormalities seen in people with CP are reminiscent of central pain syndromes [4] and that the degree of sensory abnormalities correlates with pain severity [5].

Individuals with CP form a heterogeneous population, which creates unique challenges to the rigorous study of chronic pain. The degrees, distributions, and physiologic subtypes of motor impairment vary substantially—mirroring the variability in etiology and the pattern and extent of CNS involvement. There is similar variation in cognitive profiles–45% of individuals with CP have intellectual disability, and large discrepancies between cognitive domains are common [6]. Superimposed communicative difficulties are prevalent [7], as are comorbid anxiety and mood disorders [8], which have well-established associations with chronic pain in other populations [9].

With this recognition, we believe that a comprehensive understanding of chronic pain in CP requires consideration of many factors—some known to be important in other populations with chronic pain and some specific to individuals with CP. In this study, we aimed to identify clinical factors associated with specific chronic pain characteristics in communicative adults with CP. Viewing pain as a multi-dimensional phenomenon (including pain qualities and interference with functioning as well as intensity), we include comparison to typical adults in order to provide scale when illustrating the range of pain phenotypes along each dimension. Focusing on communicative adults with CP, we further aimed to identify relationships between pain-related clinical factors in hopes of (1) facilitating dimensionality reduction in future studies and (2) identifying feature clusters suggestive of specific pain mechanisms.

## Materials and Methods

### Study Design

This was a cross-sectional study of adults with CP (CP group) as well as healthy, neurotypical adult volunteers (NT group) enrolled following approval by the Johns Hopkins Medicine Institutional Review Board.

### Participants

Case group adults were purposively recruited from a clinic database at the Kennedy Krieger Institute Phelps Center for Cerebral Palsy and Developmental Medicine. Inclusion criteria for the case group included: a diagnosis of CP; ≥18 years of age; communication functional classification system (CFCS) level I–III and ability to respond unambiguously to at least 65 multiple-choice items. Participants either consented independently or assented to participate with formal consent provided by their legally authorized representative. The authors acknowledge that use of a convenience sample permits bias, for example, in selecting for participants receiving ongoing medical surveillance. We attempted to mitigate bias (1) by maintaining wide inclusion criteria (e.g., permitting individuals with mild or severe motor impairment to participate) so as to capture a wide range of variability and (2) by restricting participation to individuals able to self-report for key outcome measures as observational pain measures may not be accurate for individuals with CP [10].

Neurotypical (NT) participants ≥18 of age were recruited as volunteers *via* flyers distributed through peer networks. Potential control participants were required not to have neurological or developmental diagnoses or a history of premature birth. Medical diagnoses of migraine (*n* = 1) and degenerative disk disease (*n* = 2) were not considered to be exclusionary given their high prevalence in the general population [15% [11] and ~75% by age 50 [12], respectively].

### Data Collection

Data collection included clinical information obtained from participant/caregiver report and available medical records as well as from a structured in-person interview and neurological examination performed by clinical providers (authors EMC and CL) with expertise evaluating individuals with neurodevelopmental disabilities. Nearly all participants elected to complete assessments in a single 1–2 h evaluation. However, as missing data may lead to bias, we permitted individuals to split assessment into multiple sessions if needed and compensated participants $20 per study visit. Two participants with CP elected to split assessment into two sessions; descriptively, neither participant reported qualitative changes in medical or pain status in the interim. As such, we considered data collected to represent a single point in time for any particular participant—in keeping with analysis using cross-sectional methodology. The number of cases reported here represent interval analysis of in-person evaluations performed in Year 1 of ongoing study.

Primary outcome measures for this study were self-reported pain intensity, pain qualities (nociceptive and neuropathic), and interference from pain with common activities as assessed using standardized PROMIS [13] questionnaires. PROMIS summary scores are reported as T-scores (population mean score of 50; standard deviation of 10) with respect to currently-available US adult norms (PROMIS Wave 1 for pain intensity and pain interference [14]; PROMIS Wave 2 for pain quality).

Other pain characteristics were also evaluated using standardized instruments. Description of typical pain sites was assessed using the Swedish CPUP pain location instrument [15]. Pain catastrophizing thoughts were assessed using the Pain Catastrophizing Scale [16]. PainDETECT-Q [17] was administered as an additional measure of neuropathic pain characteristics. Three specific PainDETECT-Q items relate to allodynia symptoms (items involving pain to “light touching,” “cold or heat,” or to “slight pressure”) and were used to assess frequency of allodynia.

Additional domains of interest (demographic factors, etiology, cognitive/affective factors, functional status, and somatosensory and motor characteristics) were assessed using additional standardized questionnaire instruments and by structured physical examination (Table 1). Further details regarding covariates of interest are reported in the Appendix.

**Table 1:** Summary of data elements included in analysis.

**Data elements [#]**	**Included data elements**
PROMIS short forms [4]	Pain intensity (Short Form 3a), pain interference (Short Form 4a), nociceptive quality, neuropathic quality (Short Form 5a) T-scores
PainDETECT [4]	Total score and scores on mechanical, temperature, and pressure allodynia items
CPUP pain location [11]	Head, neck, back, shoulders, arms/hands, hips, knees, feet, teeth, stomach, skin/pressure pain ratings
Demographics [4]	Age in years, gender identification, years of education completed, income range
CP functional rating scales [3]	GMFCS E&R, MACS, CFCS
Etiology—neuroimaging findings [3]	Binarized factors: Presence of white matter injury; presence of basal ganglia/thalamic injury; presence of a malformation
Etiology—gestational age at birth [1]	In completed weeks
Orthopedic surgical history [2]	Number of total orthopedic surgical events, maximal orthopedic surgical invasiveness
Current medication usage [4]	Binarized factors: Currently taking scheduled pain medication; currently taking tone-modifying medication; currently taking oral tone-modifying medication; intrathecal baclofen pump currently in use
Cognitive/affective diagnosis history [7]	Diagnosis of intellectual disability; diagnosis of attention deficit/hyperactivity disorder; diagnosis of anxiety; diagnosis of a mood disorder; verbal cognitive standard score; non-verbal cognitive standard score; attention/working memory standard score
Current anxiety/depression symptoms [1]	PHQ-4 total score
Pain catastrophizing symptoms [4]	PCS total score as well as rumination, magnification, and helplessness subscores
Perceived stress [1]	PSS total score
Insomnia [1]	ISI total score
Neurologic exam—mental status [1]	Attention/working memory score
Neurologic exam—somatosensory exam [7]	Mechanical detection threshold *via* Von Frey; presence of focal sharpness sensory abnormality; presence of focal thermal sensory abnormality; presence of a vibratory sensory abnormality; number of proprioception items correct; number of stereognosis items correct; tactile discrimination threshold *via* JVP domes
Neurologic exam—motor exam [4]	Presence of spasticity and/or dystonia on HAT; four-extremity summed MAS; whole-body BADS score

*PROMIS, Patient-Reported Outcomes Measurement Information System; GMFCS E&R, Gross Motor Functional Classification System, Expanded and Revised; MACS, Manual Ability Classification System; CFCS, Communication Functional Classification System; PHQ-4, Patient Health Questionnaire-4; PCS, Pain Catastrophizing Scale; PSS, Perceived Stress Scale; ISI, Insomnia Severity Index (31); JVP, Johnson-Van Boven-Philips; HAT, Hypertonia Assessment Tool (32); MAS, Modified Ashworth Scale score (33); BADS, Barry-Albright Dystonia Scale (34)*.

Statistics were performed in MATLAB 2019a (MathWorks, Natick, MA). Pain outcome measures were described *via* inter-group (CP vs. NT) comparisons. We utilized statistical measures that do not assume normally-distributed data (effect size described in terms of differences between group medians and hypothesis testing *via* two-sided non-parametric ranksum tests).

Associations between PROMIS pain T-scores and covariates were examined within the CP cohort only. For ease of interpretation, we report association strengths in terms of percent of variance shared. We selected a statistic (Spearman's ρ·|ρ|) that does not assume a linear relationship between variables and preserves a positive/negative sign indicating the direction of correlation. That is, ρ·|ρ| = 0 implies no shared variance, ρ·|ρ| = +0.5 implies a positive correlation with 50% shared variance, and ρ·|ρ| = **–**0.5 implies a negative correlation with 50% shared variance. To better evaluate relationships between somatosensory and motor variables beyond their associations with pain, a cross-covariate correlation table was also calculated. As a gross measure of statistic sensitivity, note that at *n* = 17, ρ·|ρ| reflecting >23% shared variance (positive or negative) corresponds to uncorrected *p* < 0.05 against a hypothesis of no correlation.

## Results

### Participant Characteristics

Sixty individuals with CP were screened; of these, 17 met screening criteria and were enrolled. Data from all enrolled individuals were analyzed. Sixty-two data elements included in the final analysis, and NT participants (*n* = 10) completed all items. Participants with CP (*n* = 17) completed most items [median participant completion rate 87%; Interquartile range (IQR) 74–94%]. Reasons cited for incomplete data include participant fatigue, scheduling difficulties, and inability to comprehend selected questions/tasks. Factors correlating most strongly with more missing data were high CFCS (ρ·|ρ| = +0.68), high Modified Ashworth Scale scores (ρ·|ρ| = +0.56), and high Gross Motor Functional Classification System, Expanded and Revised (GMFCS E&R; ρ·|ρ| = +0.46). (Tables 1, 2 and Supplementary Tables 1, 2).

**Table 2:** Cohort pain, somatosensory, and cognitive/affective characteristics.

	**Participants with cerebral palsy**				**Neurotypical controls**				***P*-value for groupwise difference**
	**# (%)**	**Median ± IQR**	**Range**	**Data available**	**# (%)**	**Median ± IQR**	**Range**	**Data available**	
**Pain characteristics**									
PROMIS Pain Interference T-score (higher = more interference)		50.2 ± 10.1	41.6–66.3	17/17		41.6 ± 6.5	41.6–61.3	10/10	0.11
PROMIS Pain Intensity T-score (higher = more pain)		43.8 ± 7.4	30.7–56.3			30.7 ± 8.6	30.7–56.3		0.027
PROMIS Nociceptive pain T-score (higher = more pain)		43.2 ± 7.8	30.3–53.2			37.3 ± 9.8	30.3–56.5		0.034
PROMIS Neuropathic pain T-score (higher = more pain)		42.1 ± 9.2	37.2–63.4			37.2 ± 0.0	37.2–55.5		0.013
PainDETECT Neuropathic Pain Score (higher = more pain)		4.0 ± 8.5	−1–29	15/17		1.0 ± 3.0	1–11	10/10	0.061
**Somatosensory characteristics**									
Von Frey light touch detection threshold (gm; geometric mean of four extremities)		0.57± 0.41	0.07–0.89	13/17		0.21± 0.37	0.11–0.89	10/10	0.43
Vibratory sensory abnormality (either hand)	3/12 (25%)			12/17	3/10 (30%)			10/10	0.83
Proprioception items correct (10 trials/hand)		19.0 ± 3.0	13–20	11/17		20.0 ± 0.0	20–20	10/10	0.0037
Stereognosis items correct (5 trials/hand)		9.0 ± 3.0	4–10	13/17		10.0 ± 0.0	10–10	10/10	0.0086
JVP tactile discrimination threshold (mm; geometric mean of two hands; higher = less sensitive)		4.7± 6.9	1.2–12	11/17		1.7 ± 0.48	1.4–2.4	10/10	0.015
Sharp sensory abnormality (any extremity)	6/12 (50%)			12/17	2/10 (20%)			10/10	0.17
Cool sensory abnormality (any extremity)	6/12 (50%)			12/17	3/10 (30%)			10/10	0.37
**Cognitive/affective factors**									
PHQ-4 total score (anxiety and depression; higher = more symptoms)		0.0 ± 2.0	0–6	13/17		0.0 ± 0.0	0–1	10/10	0.13
Perceived stress scale total score (higher = more symptoms)		12.0 ± 13.8	2–24	10/17		3.5 ± 5.3	0–17	10/10	0.030
Pain catastrophizing scale- total (higher = more symptoms)		8.0 ± 4.3	2–34	12/17		2.5 ± 5.5	0–18	10/10	0.027
-Rumination Subscore		3.0 ± 4.3	0–14			1.5 ± 2.8	0–5		0.28
-Magnification subscore		2.0 ± 2.3	0–5			1.0 ± 1.8	0–3		0.073
-Helplessness subscore		3.0 ± 4.0	0–15			0.0 ± 1.0	0–10		0.023
Attention/working memory screen score (higher = better performance)		1.0 ± 4.0	0–4	15/17		4.0 ± 0.8	1–4	10/10	0.028
Insomnia severity (higher = more symptoms)		5.0 ± 3.0	0–13	9/17		3.0 ± 3.5	0–9	10/10	0.20

*Self-reported pain ratings as well as associated somatosensory and cognitive/affective factors were assessed via in-person interview and physical exam including multimodal somatosensory testing*.

*IQR, interquartile range; PROMIS, patient-reported outcomes measurement information system; JVP, Johnson/Van Boven/Philips; PHQ-4, patient health questionnaire-4*.

Both study groups primarily consisted of young adults (Median ± IQR age 26y8m ± 7y3m for individuals with CP and 29y7m ± 6y2m for typical individuals, respectively; between-group *p* = 0.35 by ranksum test). Individuals with CP were approximately evenly divided by gender (47% female), while NT participants were predominantly female (90%; *p* = 0.04). Most individuals in both groups completed high school as well as additional education (Median ± IQR 14.3 ± 4.0 years for individuals with CP and 18.5 ± 4.0 years for typical individuals, respectively; *p* = 0.02). Annual household income varied from < $15,000 to >$100,000 in individuals with CP; most NT individuals were living alone and not yet making an income (Supplementary Table 1). Individuals with CP reported higher perceived stress (Median ± IQR PSS scores 12.0 ± 13.8 vs. 3.5 ± 5.3; *p* = 0.030; Table 2 and Supplementary Table 1).

Individuals with CP included widely-varying gross motor (GMFCS E & R I-V) and fine motor (MACS I–IV) skills. Sixty-seven percent were born preterm (at median ± IQR gestational age 31.0 ± 11.5 weeks). All individuals with records of clinical neuroimaging demonstrated apparent abnormalities (93% involving subcortical white matter; 33% involving basal ganglia and/or thalami; 8.5% with a cortical malformation noted). All participants who underwent physical exam demonstrated a degree of spasticity, and nearly all (10/11) demonstrated dystonia detectable on the Barry-Albright Dystonia Scale. Fifteen of seventeen individuals had undergone orthopedic surgery; of the fifteen for which a comprehensive history could be obtained, 40% had had spinal surgery/hip reconstruction (Grade 3), 20% had had other bony surgeries only (Grade 2), and 27% had had soft tissue procedures only (Grade 1; Supplementary Table 2).

Two participants with CP were taking pain medications on a scheduled basis (gabapentin and celecoxib, respectively), but a majority (82%) were receiving tone-modulating medications (47% oral systemic medications only; 29% intrathecal baclofen only; 6% both; Supplementary Table 2).

No NT participants had detectable spasticity or dystonia or were taking tone-modulating medication. Two were taking scheduled pain medication (gabapentin for degenerative disk disease and arthritis; and nortriptyline for migraine, respectively). No individuals in either group had sleep-related symptoms in the range of clinical insomnia (ISI scores >14; Table 2).

A minority of individuals with CP had intellectual disability (24%) or ADHD (6%). Median performance on the study visit attention/working memory screen was 1 item correct out of 4 as compared to a median of 4 items correct out of 4 for typical participants (*p* = 0.016 against a null hypothesis of no group difference; Supplementary Table 2).

Several individuals with CP had anxiety (24%) or mood disorders (18%). In contrast, no NT participants had anxiety, though two had mood disorders (20%). Current affective symptoms as measured by PHQ-4 were minimal in both groups (median score 0; Table 2 and Supplementary Table 2).

### Pain Characteristics

Individuals with CP reported higher pain intensity (Median ± IQR PROMIS T-scores 43.8 ± 7.4 vs. 30.7 ± 8.6; *p* = 0.03). Greater interference from pain on activities did not reach significance (*p* = 0.11), though median difference (Median ± IQR PROMIS T-scores 50.2 ± 10.1 vs. 41.6 ± 6.5) exceeds the estimated minimally important difference of 2–4 points seen in other chronic pain conditions [[18]; Table 2].

Individuals in the CP cohort reported greater nociceptive pain characteristics (Median ± IQR PROMIS T-scores 43.2 ± 7.8 vs. 37.3 ± 9.8; *p* = 0.03) as well as neuropathic pain characteristics (Median ± IQR PROMIS T-scores 42.1 ± 9.2 vs. 37.2 ± 0.0; *p* = 0.01). Of 15 individuals with CP who completed the PainDETECT instrument that includes items on allodynia, 7 (47%) reported a degree of allodynia to one or more modalities (13% to light touch; 27% to warm or cool stimuli; 33% to light pressure). In contrast, only pressure allodynia was reported by NT participants (20%; Table 2).

The most common site of pain in participants with CP was the back (71%) followed by knees (53%), hips, shoulders, and neck (all 41%). In contrast, typical participants most commonly reported head pain (60%) or back pain (40%).

### Correlates of Chronic Pain

**Figure 1:**
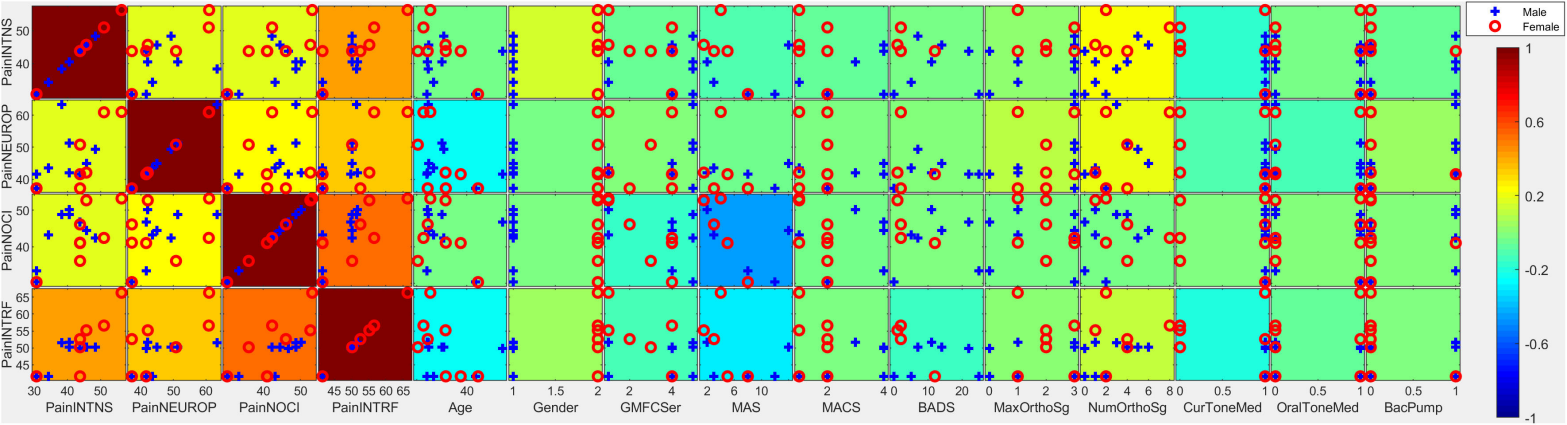
Covariates of pain intensity. Individual subtiles indicate scatterplots between pain factors (rows) and covariates (columns). Background color indicates the strength and direction of correlation (ρ·|ρ|). Signs for individual covariates are aligned such that higher values code higher levels of dysfunction with the following exceptions: higher age is coded as higher; female is coded as higher; taking medication is coded as higher; less education is coded as higher; lower household income is coded as higher; lower gestational age is coded as higher. Categorical variables with all but ≤ 3 individuals scoring in one category were excluded from this analysis due to lack of power. PainINTNS, PROMIS Pain Intensity T-score; PainNEUROP, PROMIS Neuropathic Pain Quality T-score; PainNOCI, PROMIS Nociceptive Pain Quality T-score; PainINTRF, PROMIS Pain Interference T-score; GMFCSer, Gross Motor Functional Classification System, Expanded and Revised; MAS, 4-extremity summed Modified Ashworth Scale score; MACS, Manual Ability Classification System; BADS, Total Barry-Albright Dystonia Scale score; MaxOrthoSg, score indicating most invasive orthopedic surgery undergone; NumOrthoSg, total number of orthopedic surgical events to date; CurToneMed, Currently taking oral/intrathecal tone-modulating medication (binarized); OralToneMed, Currently taking oral tone-modulating medication (binarized); BacPump, Intrathecal baclofen pump currently in use; YrEdu, Years of Education completed (high school = 12); CFCS, Communication Functional Classification System; IDdx, presence of intellectual disability diagnosis (binarized); AttWM, Attention/Working memory screen score; ANXdx, presence of an anxiety disorder diagnosis (binarized); PHQ4tot, Patient Health Questionnaire-4 total score; PCStot, Pain Catastrophizing Scale total score; PSStot, Perceived Stress Scale total score; IncRng, Household income range; VFthresh, Von Frey light touch sensation threshold; VibAbn, Vibratory sensation abnormality (binarized); propSum, Proprioception score; StereoSum, Stereognosis score; JVPthresh, Johnson-Van Boven-Philips dome tactile discrimination threshold; SharpAbn, Sharp sensation abnormality (binarized); CoolAbn, Cool sensation abnormality (binarized); GestAge, Gestational age at birth (weeks); BGTinj, presence of clinically-identified basal ganglia or thalamic injury on neuroimaging (binarized); blanks, percent data missingness.

**Figure 2:**
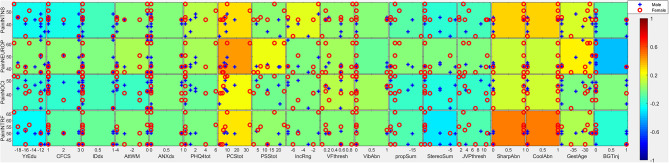
Covariates of pain intensity. Individual subtiles indicate scatterplots between pain factors (rows) and covariates (columns). Background color indicates the strength and direction of correlation (ρ·|ρ|). Signs for individual covariates are aligned such that higher values code higher levels of dysfunction with the following exceptions: higher age is coded as higher; female is coded as higher; taking medication is coded as higher; less education is coded as higher; lower household income is coded as higher; lower gestational age is coded as higher. Categorical variables with all but ≤ 3 individuals scoring in one category were excluded from this analysis due to lack of power. PainINTNS, PROMIS Pain Intensity T-score; PainNEUROP, PROMIS Neuropathic Pain Quality T-score; PainNOCI, PROMIS Nociceptive Pain Quality T-score; PainINTRF, PROMIS Pain Interference T-score; GMFCSer, Gross Motor Functional Classification System, Expanded and Revised; MAS, 4-extremity summed Modified Ashworth Scale score; MACS, Manual Ability Classification System; BADS, Total Barry-Albright Dystonia Scale score; MaxOrthoSg, score indicating most invasive orthopedic surgery undergone; NumOrthoSg, total number of orthopedic surgical events to date; CurToneMed, Currently taking oral/intrathecal tone-modulating medication (binarized); OralToneMed, Currently taking oral tone-modulating medication (binarized); BacPump, Intrathecal baclofen pump currently in use; YrEdu, Years of Education completed (high school = 12); CFCS, Communication Functional Classification System; IDdx, presence of intellectual disability diagnosis (binarized); AttWM, Attention/Working memory screen score; ANXdx, presence of an anxiety disorder diagnosis (binarized); PHQ4tot, Patient Health Questionnaire-4 total score; PCStot, Pain Catastrophizing Scale total score; PSStot, Perceived Stress Scale total score; IncRng, Household income range; VFthresh, Von Frey light touch sensation threshold; VibAbn, Vibratory sensation abnormality (binarized); propSum, Proprioception score; StereoSum, Stereognosis score; JVPthresh, Johnson-Van Boven-Philips dome tactile discrimination threshold; SharpAbn, Sharp sensation abnormality (binarized); CoolAbn, Cool sensation abnormality (binarized); GestAge, Gestational age at birth (weeks); BGTinj, presence of clinically-identified basal ganglia or thalamic injury on neuroimaging (binarized); blanks, percent data missingness.

Reported pain intensity, pain interference, and neuropathic and nociceptive qualities were closely correlated in individuals with CP (ρ·|ρ| between constructs ranging from +0.19 to +0.55) (see Figures 1, 2 for correlation plots). Higher pain intensity was associated with female gender (ρ·|ρ| = 0.16), more symptoms of anxiety/depression on PHQ-4 (ρ·|ρ| = +0.10), more pain catastrophizing symptoms (total PCS score; ρ·|ρ| = +0.31) and lower household income (ρ·|ρ| = −0.19).

Individuals with greater motor impairment did not report more pain. Higher GMFCS E&R was associated with lower pain intensity (ρ·|ρ| = −0.05), pain interference (ρ·|ρ| = −0.12), and nociceptive pain qualities (ρ·|ρ| = −0.14). Similar patterns were seen with higher MAS (ρ·|ρ| = −0.10, −0.28, and −0.44, respectively). In contrast, a higher number of prior orthopedic surgeries was associated with higher pain intensity and pain impairment (ρ·|ρ| = +0.24 and +0.13, respectively). Current use of tone-altering medication was associated with lower pain intensity (ρ·|ρ| = −0.16) and pain interference (ρ·|ρ| = −0.19).

Cognitive/communicative impairments were associated with less reported pain. Higher CFCS was associated with lower pain intensity (ρ·|ρ| = −0.21), pain interference (ρ·|ρ| = −0.07), and nociceptive pain qualities (ρ·|ρ| = −0.16). A similar pattern was seen in association with a diagnosis of intellectual disability (ρ·|ρ| = −0.12,−0.05, and −0.12, respectively), and higher educational attainment was associated with greater reported pain (ρ·|ρ| = +0.23, +0.25, and +0.15, respectively). Better attention/working memory function was also associated with more nociceptive pain qualities (ρ·|ρ| = +0.13).

Somatosensory associations with chronic pain in individuals with CP were complex. Higher pain intensity was associated with the presence of qualitative abnormalities in sharp/cool sensation (ρ·|ρ| = +0.33). In contrast, higher pain intensity, pain interference, and nociceptive qualities were associated with better performance on cortical sensory tasks such as proprioception (ρ·|ρ| = +0.08, +0.10, and +0.14, respectively), stereognosis (ρ·|ρ| = +0.17, +0.29, and +0.19, respectively), and tactile discrimination *via* JVP domes (ρ·|ρ| = −0.12, −0.10, and −0.15, respectively; high thresholds represent poor discrimination).

### Relationships Between Correlates

**Figure 3:**
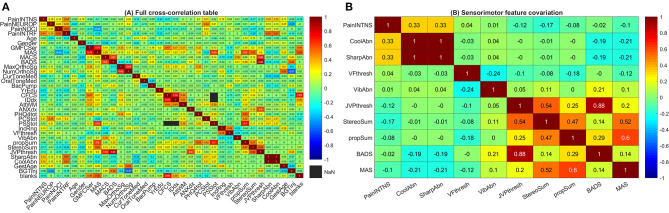
Cross-correlation tables. **(A)** Full cross-correlation table. This heatmap presents covariation (ρ·|ρ|) between pain factors, somatosensory factors, and motor factors. Coding for each variable is as in Figure 1 with signs inverted (preserving rank) for some dimensions (proprioception, stereognosis, and attention/working memory scores as well as years of education attained, household income, and gestational age at birth) such that higher scores correspond to greater deficits or predicted risk. **(B)** Sensorimotor feature covariation. This heatmap presents covariation (ρ·|ρ|) between pain intensity, somatosensory factors, and motor factors. For ease of visualization, signs for individual covariates are aligned such that higher values code for greater degrees of abnormality. Note that clusters emerge with sharp and cool sensation correlating with pain intensity (top left corner) as well as proprioception and cortical sensory modalities correlating with motor dysfunction (bottom right corner). PainINTNS, PROMIS Pain Intensity T-score; PainNEUROP, PROMIS Neuropathic Pain Quality T-score; PainNOCI, PROMIS Nociceptive Pain Quality T-score; PainINTRF, PROMIS Pain Interference T-score; GMFCSer, Gross Motor Functional Classification System, Expanded and Revised; MAS, 4-extremity summed Modified Ashworth Scale score; MACS, Manual Ability Classification System; BADS, Total Barry-Albright Dystonia Scale score; MaxOrthoSg, score indicating most invasive orthopedic surgery undergone; NumOrthoSg, total number of orthopedic surgical events to date; CurToneMed, Currently taking oral/intrathecal tone-modulating medication (binarized); OralToneMed, Currently taking oral tone-modulating medication (binarized); BacPump, Intrathecal baclofen pump currently in use; YrEdu, Years of Education completed (high school = 12); CFCS, Communication Functional Classification System; IDdx, presence of intellectual disability diagnosis (binarized); AttWM, Attention/Working memory screen score; ANXdx, presence of an anxiety disorder diagnosis (binarized); PHQ4tot, Patient Health Questionnaire-4 total score; PCStot, Pain Catastrophizing Scale total score; PSStot, Perceived Stress Scale total score; IncRng, Household income range; VFthresh, Von Frey light touch sensation threshold; VibAbn, Vibratory sensation abnormality (binarized); propSum, Proprioception score; StereoSum, Stereognosis score; JVPthresh, Johnson-Van Boven-Philips dome tactile discrimination threshold; SharpAbn, Sharp sensation abnormality (binarized); CoolAbn, Cool sensation abnormality (binarized); GestAge, Gestational age at birth (weeks); BGTinj, presence of clinically-identified basal ganglia or thalamic injury on neuroimaging (binarized); blanks, percent data missingness.

Somatosensory, motor, and cognitive features were not independent. Motor findings were closely associated—particularly the association between GMFCS E&R and MAS (ρ·|ρ| = +0.74) and that between MACS and BADS (ρ·|ρ| = +0.60). However, motor impairment was also closely associated with cognitive impairment (e.g., association between GMFCS E&R and a diagnosis of intellectual disability had ρ·|ρ| = +0.37) and with cortical sensory impairment (e.g., association between MACS and JVP dome tactile discrimination threshold with ρ·|ρ| = −0.68; Figure 3).

Associations with etiologic factors similarly cut across domains. Prematurity (inverse gestational age) was associated with greater pain intensity (ρ·|ρ| = +0.17), pain interference (ρ·|ρ| = +0.09), and neuropathic pain features (ρ·|ρ| = +0.27) but also with greater MAS (ρ·|ρ| = +0.25), greater number (ρ·|ρ| = +0.52) and maximal severity of orthopedic surgeries (ρ·|ρ| = +0.30), poorer attention/working memory scores (ρ·|ρ| = −0.11), with an anxiety diagnosis (ρ·|ρ| = +0.11), and lower household income (ρ·|ρ| = −0.29). Associated somatosensory features include sharp/cool abnormalities (ρ·|ρ| = +0.06; Figure 3).

Basal ganglia/thalamic injury were associated with term birth (ρ·|ρ| = +0.33); additional associations outside of those reported for gestational age include higher dystonia scores (ρ·|ρ| = +0.48) and tactile discrimination deficits (ρ·|ρ| = +0.33; Figure 3).

## Discussion

In this preliminary investigation of adults with CP, our data supports the established finding that chronic pain is prominent in this population and that it can have substantive effects on quality of life [1]. Mirroring the variability of CP sensorimotor phenotypes, chronic pain is equally complex and includes prominent neuropathic as well as nociceptive components, as reported by other investigators [3].

As seen in other populations with chronic pain, several large effect-size associations with pain intensity are apparent even in this small sample. Associations seen in other chronic pain conditions including female gender [19], symptoms of anxiety/mood disorders [20], pain catastrophizing symptoms [21], and low socioeconomic status [22] appear to apply to adults with CP as well.

Approaching chronic pain from a brain circuitry perspective, hints emerge of important somatosensory factors that are more specific to individuals with CP. Somatosensory features cluster grossly into spinothalamic signs (sharp/temperature sensation) and cortical sensory signs (tactile discrimination, stereognosis, and proprioception). Of these, spinothalamic deficits were positively correlated with higher pain ratings in individuals with CP. Fully quantitative sensory testing is needed to clarify the relationship between pain and somatosensory sensitivity in these individuals.

An analogous concurrence of chronic pain with spinothalamic sensory alterations occurs in post-stroke central pain syndromes (previously known as the “thalamic syndrome”)—which follow injury to subcortical ascending pathways (from the spinothalamic tract to the thalamus to thalamocortical projections) [23]. In contrast, the confluence of cortical sensory signs and motor deficits suggests a basis in somatosensory and motor cortex [24, 25]. The presence of strong associations along neuroanatomical lines suggests a role for quantitative neuroimaging in correlating imaging patterns with specific patterns of sensorimotor deficits—particularly in the setting of recognized abnormalities involving cortical and thalamocortical organization in CP-related perinatal brain injury [26–28].

Unexpectedly, a greater severity of motor deficits (as measured by GMFCS E&R or by physiologic measures of spasticity) in this study was associated with lower pain intensity (particularly nociceptive pain) and less pain interference. The association of decreased reported pain with lower communicative and cognitive functioning (even within this cohort of individuals able to self-report) suggest a need for caution and further study as it is not clear whether this effect reflects decreased pain, decreased ability to communicate pain, or limited sensitivity of established pain instruments when used for these individuals. More detailed evaluation of the effects of specific cognitive factors is needed—as is the need to cross-validate pain measures for individuals with cognitive/communicative limitations.

Limited etiologic information is available, but factors appear to show cross-domain patterns of associated deficits in this population. Associations with lower gestational age at birth, for example, recapitulate established manifestations of encephalopathy of prematurity (EoP) including attention deficits, anxiety, and, in this population, spasticity [29]. It is possible that additional pain and sensory associations seen here (specifically, with greater neuropathic pain and spinothalamic sensory abnormalities) are previously under-recognized manifestations of a severe EoP phenotype. However, this study is unable to separate prematurity from secondary associations such as early painful procedure burden, which have been associated with long-term somatosensory and neurodevelopmental responses [30]. As the study group only includes individuals with CP, this study also cannot separate the effects of term birth from those of causes of CP that are more common in children born full-term (e.g., hypoxic-ischemic encephalopathy). From this perspective, positive associations between term birth, basal ganglia/thalamic abnormalities (commonly impacted by perinatal hypoxia-ischemia), and higher dystonia scores (commonly seen following basal ganglia injury) are expected. We posit that lower associated pain ratings are then a consequence of “less prematurity” rather than a protective effect of basal ganglia/thalamic injury.

We recognize that this study was exploratory, and our study cohorts represent small convenience samples with non-negligible missing values. As such, sampling in individuals with CP is adequate across the span of some variables (e.g., GMFCS E&R) but not others (e.g., age and communication status)—limiting the generalizability of findings. Widespread and varied medication usage within the case group may further impact pain ratings. This study was only powered to search for large effects—not to detect smaller effects, exclude associations, or to disentangle mediating/moderating effects. The control group used is an imperfect comparison; the two groups share similar age characteristics but different gender distributions and educational backgrounds. That said, control group data was only used in inter-group comparisons and does not affect discussion of associations within the CP group.

Recognizing these limitations, we believe that similar multimodal approaches will provide further understanding of the phenotype of chronic pain and that correlates of interest identified here should be evaluated in larger-scale descriptive and mechanistic investigations of chronic pain in people with CP.

## Data Availability Statement

The raw data supporting the conclusions of this article will be made available by the authors, without undue reservation.

## Ethics Statement

The studies involving human participants were reviewed and approved by Johns Hopkins IRB. The patients/participants provided their written informed consent (or assent with legally authorized representative's consent) to participate in this study.

## Author Contributions

EC, AH, and SR conceived the study. EC, CL, CC, ES, AH, and SR contributed to study design. EC and CL contributed to data acquisition. EC, CL, and AH had full access to data supporting this manuscript. EC, XY, and SR contributed to data analysis. All authors contributed to interpretation of data and approval of the final manuscript.

## Conflict of Interest

The authors declare that the research was conducted in the absence of any commercial or financial relationships that could be construed as a potential conflict of interest.

## References

[1] Vogtle LK. Pain in adults with cerebral palsy: impact and solutions. Dev Med Child Neurol. (2009) 51(Suppl 4):113–21. 10.1111/j.1469-8749.2009.03423.x 19740218

[2] Blackman JA Svensson CI Marchand S. Pathophysiology of chronic pain in cerebral palsy: implications for pharmacological treatment and research. Dev Med Child Neurol. (2018) 60:861–65. 10.1111/dmcn.13930 29882358

[3] Russo RN Miller MD Haan E Cameron ID Crotty M. Pain characteristics and their association with quality of life and self-concept in children with hemiplegic cerebral palsy identified from a population register. Clin J Pain. (2008) 24:335–42. 10.1097/ajp.0b013e318162eae0 18427232

[4] Blankenburg M Junker J Hirschfeld G Michel E Aksu F Wager J. Quantitative sensory testing profiles in children, adolescents and young adults (6–20 years) with cerebral palsy: hints for a neuropathic genesis of pain syndromes. Eur J Paediatr Neurol. (2018) 22:470–81. 10.1016/j.ejpn.2017.12.015 29337004

[5] Riquelme I Cifre I Montoya P. Age-related changes of pain experience in cerebral palsy and healthy individuals. Pain Med. (2011) 12:535–45. 10.1111/j.1526-4637.2011.01094.x 21463475

[6] Reid SM Meehan EM Arnup SJ Reddihough DS. Intellectual disability in cerebral palsy: a population-based retrospective study. Dev Med Child Neurol. (2018) 60:687–94. 10.1111/dmcn.13773 29667705

[7] Sigurdardottir S Vik T. Speech, expressive language, and verbal cognition of preschool children with cerebral palsy in Iceland. Dev Med Child Neurol. (2011) 53:74–80. 10.1111/j.1469-8749.2010.03790.x 21039439

[8] Whitney DG Warschausky SA Ng S Hurvitz EA Kamdar NS Peterson MD. Prevalence of mental health disorders among adults with cerebral palsy: a cross-sectional analysis. Ann Intern Med. (2019) 171:328–33. 10.7326/M18-3420 31382276 PMC9704040

[9] Dersh J Polatin PB Gatchel RJ. Chronic pain and psychopathology: research findings and theoretical considerations. Psychosom Med. (2002) 64:773–86. 10.1097/00006842-200209000-00010 12271108

[10] Fox MA Ayyangar R Parten R Haapala HJ Schilling SG Kalpakjian CZ. Self-report of pain in young people and adults with spastic cerebral palsy: interrater reliability of the revised face, legs, activity, cry, and consolability (r-FLACC) scale ratings. Dev Med Child Neurol. (2019) 61:69–74. 10.1111/dmcn.13980 30051908

[11] Steiner TJ Stovner LJ Birbeck GL. Migraine: the seventh disabler. J Headache Pain. (2013) 14:1. 10.1186/1129-2377-14-1 23566305 PMC3606966

[12] Teraguchi M. Prevalence and distribution of intervertebral disc degeneration over the entire spine in a population-based cohort: the wakayama spine study. Spine J. (2013) 13:S83. 10.1016/j.spinee.2013.07.229 24239943

[13] Cella D Yount S Rothrock N Gershon R Cook K Reeve B. The patient-reported outcomes measurement information system (PROMIS): progress of an NIH roadmap cooperative group during its first two years. Med Care. (2007) 45:S3–11. 10.1097/01.mlr.0000258615.42478.55 17443116 PMC2829758

[14] Cella D Riley W Stone A Rothrock N Reeve B Yount S. The patient-reported outcomes measurement information system (PROMIS) developed and tested its first wave of adult self-reported health outcome item banks: 2005–2008. J Clin Epidemiol. (2010) 63:1179–94. 10.1016/j.jclinepi.2010.04.011 20685078 PMC2965562

[15] Westbom L Rimstedt A Nordmark E. Assessments of pain in children and adolescents with cerebral palsy: a retrospective population-based registry study. Dev Med Child Neurol. (2017) 59:858–63. 10.1111/dmcn.13459 28509356

[16] Sullivan MJL Bishop SR Pivik J. The pain catastrophizing scale: development and validation. Psychol Assess. (1995) 7:524–32. 10.1037//1040-3590.7.4.524

[17] Freynhagen R Tölle TR Gockel U Baron R. The painDETECT project—far more than a screening tool on neuropathic pain. Curr Med Res Opin. (2016) 32:1033–57. 10.1185/03007995.2016.1157460 26907456

[18] Chen CX Kroenke K Stump TE Kean J Carpenter JS Krebs EE. Estimating minimally important differences for the PROMIS pain interference scales: results from 3 randomized clinical trials. Pain. (2018) 159:775–82. 10.1097/j.pain.0000000000001121 29200181 PMC5860950

[19] Fillingim RB King CD Ribeiro-Dasilva MC Rahim-Williams B Riley JL III. Sex, gender, and pain: a review of recent clinical and experimental findings. J Pain. (2009) 10:447–85. 10.1016/j.jpain.2008.12.001 19411059 PMC2677686

[20] McWilliams LA Cox BJ Enns MW. Mood and anxiety disorders associated with chronic pain: an examination in a nationally representative sample. Pain. (2003) 106:127–33. 10.1016/S0304-3959(03)00301-4 14581119

[21] Quartana PJ Campbell CM Edwards RR. Pain catastrophizing: a critical review. Expert Rev Neurother. (2009) 9:745–58. 10.1586/ern.09.34 19402782 PMC2696024

[22] Dahlhamer J Lucas J Zelaya C Nahin R Mackey S DeBar L. Prevalence of chronic pain and high-impact chronic pain among adults—United States, 2016. MMWR Morb Mortal Wkly Rep. (2018) 67:1001–6. 10.15585/mmwr.mm6736a2 30212442 PMC6146950

[23] Widerström-Noga E Loeser JD Jensen TS Finnerup NB. AAPT diagnostic criteria for central neuropathic pain. J Pain. (2017) 18:1417–26. 10.1016/j.jpain.2017.06.003 28666966

[24] Hsiao S. Central mechanisms of tactile shape perception. Curr Opin Neurobiol. (2008) 18:418–424. 10.1016/j.conb.2008.09.001 18809491

[25] Delhaye BP Long KH Bensmaia SJ. Neural basis of touch and proprioception in primate cortex. Compr Physiol. (2018) 8:1575–602. 10.1002/cphy.c170033 30215864 PMC6330897

[26] Scheck SM Boyd RN Rose SE. New insights into the pathology of white matter tracts in cerebral palsy from diffusion magnetic resonance imaging: a systematic review. Dev Med Child Neurol. (2012) 54:684–96. 10.1111/j.1469-8749.2012.04332.x 22646844

[27] Hoon AH Jr Stashinko EE Nagae LM Lin DDM Keller J Bastian A. Sensory and motor deficits in children with cerebral palsy born preterm correlate with diffusion tensor imaging abnormalities in thalamocortical pathways. Dev Med Child Neurol. (2009) 51:697–704. 10.1111/j.1469-8749.2009.03306.x 19416315 PMC2908264

[28] Coq J-O Strata F Russier M Safadi FF Merzenich MM Byl NN. Impact of neonatal asphyxia and hind limb immobilization on musculoskeletal tissues and S1 map organization: implications for cerebral palsy. Exp Neurol. (2008) 210:95–108. 10.1016/j.expneurol.2007.10.006 18061167

[29] Jantzie LL Robinson S. Preclinical models of encephalopathy of prematurity. Dev Neurosci. (2015) 37:277–88. 10.1159/000371721 25722056 PMC4514537

[30] Walker SM. Long-term effects of neonatal pain. Semin Fetal Neonatal Med. (2019) 24:101005. 10.1016/j.siny.2019.04.005 30987942

