# Supplementary material for: Clinical Factors Associated With Chronic Pain in Communicative Adults With Cerebral Palsy: A Cross-Sectional Study
Source: Front Pain Res (Lausanne). 2020 Nov 24;1:553026. doi: 10.3389/fpain.2020.553026 (PMC8915712; doi:10.3389/fpain.2020.553026)
Supplement: Supplementary file 1 [file Table_1.DOCX]

|  | **Participants with cerebral palsy** | | | | | **Neurotypical controls** | | | | | **p-value for groupwise difference** |
| --- | --- | --- | --- | --- | --- | --- | --- | --- | --- | --- | --- |
|  | **# (%)** | **Mean± SD** | **Median± IQR** | **Range** | **Included in Analysis** | **# (%)** | **Mean± SD** | **Median± IQR** | **Range** | **Included in Analysis** |  |
| **Female gender** | 8/17 (47%) |  |  | | 17/17 | 9/10 (90%) |  |  | | 10/10 | 0.044 |
| **Age (years/ months)** |  | 30y7m ± 9y3m | 26y8m ± 7y3m | 20y4m- 54y2m | 17/17 |  | 33y9m ± 12y9m | 29y7m ± 6y2m | 23y11m- 67y3m | 10/10 | 0.35 |
| **Years of education** |  | 14.4 ± 2.6 | 14.3 ± 4.0 | 11-19 | 16/17 |  | 17.5 ± 3.4 | 18.5 ± 4.0 | 12 - 21 | 10/10 | 0.021 |
| **Annual household income** |  |  | 25th percentile: $15,000- 24,999  Median:  $50,000- 74,999  75th percentile: $>75,000 | Minimum: <$15,000  Maximum:  >$100,000 | 11/17 |  |  | Median:  $50,000- 74,999 | Minimum: $35,000- 49,999  Maximum:  $50,000- 74,999 | 3/10 | 1.0 |

**Table S1: Cohort demographics.** Evaluation included self-reported gender, years of education (12 = completion of high school), and annual household income range (ranges from National Institutes of Neurological Disorders and Stroke recommended Common Data Elements for Cerebral Palsy). Note that household income was censored from reporting for students reported to be living alone. **Abbreviations:** IQR, Interquartile range; y, year; m, month.
