# Supplementary material for: Clinical Factors Associated With Chronic Pain in Communicative Adults With Cerebral Palsy: A Cross-Sectional Study
Source: Front Pain Res (Lausanne). 2020 Nov 24;1:553026. doi: 10.3389/fpain.2020.553026 (PMC8915712; doi:10.3389/fpain.2020.553026)
Supplement: Supplementary file 2 [file Table_2.DOCX]

|  | **# (%)** | **Median ± IQR** | **Range** | **Data available** |
| --- | --- | --- | --- | --- |
| **GMFCS E&R** |  | 4.0 ± 2.0 | 1-5 | 17/17 |
| **MACS** |  | 2.0 ± 1.5 | 1-4 | 16/17 |
| **CFCS** |  | 1.0 ± 1.3 | 1-3 | 16/17 |
| **Etiological factors** | | | |  |
| **Gestational age at birth (weeks)** |  | 31.0 ± 11.5 | 27-term | 15/17 |
| **Neuroimaging findings** | | | | |
| **Evidence of white matter involvement** | 11/12 (93%) |  | | 12/17 |
| **Evidence of basal ganglia/thalamic involvement** | 4/12 (33%) |  |  |  |
| **Evidence of brain malformation** | 1/12 (8.5%) |  |  |  |
| **Motor characteristics** | | | |  |
| **Four-extremity Modified Ashworth Scale score (higher = more severe)** |  | 4.5 ± 5.0 | 1.5-14 | 10/17 |
| **Total Barry-Albright Dystonia Scale score (higher = more severe)** |  | 7.0 ± 11.0 | 0-25 | 11/17 |
| **Orthopedic surgical history** | | | |  |
| **Number of orthopedic surgical events** |  | 2.0 ± 3.0 | 0-8 | 15/17 |
| **Highest orthopedic surgical invasiveness grade reached** |  | 2.0 ± 2.0 | 0-3 | 16/17 |
| **Ongoing scheduled medication use** | | | |  |
| **Pain medication** | 2/17 (12%) |  | | 17/17 |
| **Tone-modulating medication:Any** | 14/17 (82%) |  |  | 17/17 |
| **Enteral** | 9/17 (53%) |  |  | 17/17 |
| **Intrathecal baclofen** | 5/17 (29%) |  |  | 17/17 |
| **Cognitive/behavioral characteristics** | | | |  |
| **Intellectual disability diagnosis** | 4/17 (24%) |  | | 17/17 |
| **Attention deficit/hyperactivity disorder diagnosis** | 1/17 (5.9%) |  |  | 17/17 |
| **Anxiety disorder diagnosis** | 4/17 (24%) |  |  | 17/17 |
| **Mood disorder diagnosis** | 3/17 (18%) |  |  | 17/17 |

**Table S2: CP cohort clinical characteristics.** Etiological factors, motor characteristics, orthopedic surgical history, ongoing scheduled medication use, and cognitive/behavioral characteristics were evaluated using the medical record and individual/caregiver report with harmonization by a physician.

**Abbreviations:** IQR, interquartile range; GMFCS E&R, Gross Motor Functional Classification System, Expanded and Revised; MACS, Manual Ability Classification System; CFCS, Communication Functional Classification System.
