# Supplementary material for: Clinical Factors Associated With Chronic Pain in Communicative Adults With Cerebral Palsy: A Cross-Sectional Study
Source: Front Pain Res (Lausanne). 2020 Nov 24;1:553026. doi: 10.3389/fpain.2020.553026 (PMC8915712; doi:10.3389/fpain.2020.553026)
Supplement: Supplementary file 3 [file Table_3.DOCX]

**Online-only Appendix: Data Elements.**

Interview/exam items were selected after reviewing NINDS recommended Common Data Elements (CDEs) for CP [(1)](https://paperpile.com/c/ndoWsy/oWlLl) and designed to include associated factors established in other chronic pain conditions as well as quantifiable clinical factors relevant to CP.

Study visits were scheduled considering participant fatigue and transportation needs, and not all participants completed all testing procedures. Given limited sample size, no imputation was employed, though variables correlating strongly with missing value percentage are reported to identify potentially underrepresented subpopulations. As such, correlation analyses include all participants for which both data elements of interest are available.

Demographic factors examined included age at the time of visit, self-identified gender, years of education (completion of high school counted as 12), and household income range (NINDS CDE income ranges; range #1 indicating <$15,000/year, and #7 indicating ≥$100,000/year). Household income range was censored for individuals reporting as unemployed students living alone.

For individuals with CP, functional status was classified by reported gross motor functioning (Gross Motor Functional Classification System, Expanded and Revised; GMFCS E&R[(2)](https://paperpile.com/c/ndoWsy/qaEep)), manual ability (Manual Ability Classification System; MACS [(3)](https://paperpile.com/c/ndoWsy/8pzcW)) and communication status (CFCS). On in-person assessment, the Hypertonia Assessment Tool [(4)](https://paperpile.com/c/ndoWsy/c4v7G) was used to identify spasticity or dystonia, which when present was then quantified using the Modified Ashworth Scale (MAS) [(5)](https://paperpile.com/c/ndoWsy/kdM0v) and Barry-Albright Dystonia Scale (BADS) [(6)](https://paperpile.com/c/ndoWsy/DBb8k), respectively. For covariate analysis, single whole-body indices were generated and used as proxies for individual spasticity and dystonia severity (sum of bilateral biceps and hip adductor MAS scores (1+ counted as 1.5) and total BADS score, respectively).

Medical history included report by participants/caregivers (core and recommended CDEs above) as well as available electronic medical records. Both medical records and participant/caregiver history were considered in determination of the presence or absence of a diagnosis; in rare cases of disagreement, the examining clinical provider (authors EMC or CL in consultation with author AHH) determined whether the diagnosis in question applied. The Rosenbaum et al (7) definition was used for determining whether a participant had CP and met inclusion criteria.

Etiologic factors reported included degree of prematurity at birth as well as pattern of injury noted on extant brain imaging reports (MRI, CT, and/or neonatal ultrasound). Imaging findings were coded in a binarized manner for evidence of white matter injury (e.g. periventricular leukomalacia or sequelae of stroke), evidence of deep gray basal ganglia/thalamic injury (e.g. deep hypoxic-ischemic encephalopathy or kernicterus), and/or evidence of a brain malformation.

Cumulative “dose” of orthopedic surgical history was defined in two ways *a priori*: as total number of surgical events (distinct dates on which orthopedic surgery was performed); and using a scale to estimate the highest degree of invasiveness experienced:

3 = spinal surgery or reconstructive hip surgery

2 = no level 3 surgery, but including other bony surgery

1= no level 2-3 surgery, but including soft tissue surgery

0 = no orthopedic surgery

Binarized factors were also included for chronic, scheduled, ongoing use of pain medications and of tone-altering medications (oral systemic and/or intrathecal baclofen). Sleep disturbances were self-reported (Insomnia Severity Index [(8)](https://paperpile.com/c/ndoWsy/z1HL1)).

Cognitive/behavioral characteristics were assessed in terms of binarized presence of coexisting diagnoses (intellectual disability, attention deficit/hyperactivity disorder (ADHD), anxiety disorders, and/or mood disorders) as well as interview instruments/brief testing performed at the visit. Features of anxiety and/or depressed mood were self-reported using the Patient Health Questionnaire-4 [(9)](https://paperpile.com/c/ndoWsy/kmY9b). Participants also performed verbal digit span forwards and reversed tasks. Digit span forwards is an established sensitive measure of attention, and digit span reversed requires manipulation in working memory as well as attention. Typical adult spans forwards and reversed are approximately six and four, respectively [(10)](https://paperpile.com/c/ndoWsy/afsFo). As a screening item, individuals were administered two trials of six digits forwards and two trials of four digits reversed (one per second) and scored one point for each correct item.

Somatosensory evaluation was designed to capture many somatosensory modalities quickly and practically. Testing of several modalities relied on “bedside” clinical qualitative neurological assessments [(11)](https://paperpile.com/c/ndoWsy/CrFHe). Sharp sensation was tested with a safety pin-- sharpness of a gentle prick was elicited on the dorsum of each hand/foot in reference to the forehead. Similarly, temperature sensation in the same sites was tested using the cool surface of a 128cps tuning fork. Vibratory sensation was evaluated with a 128cps tuning fork applied to bilateral metacarpophalangeal joints of the hands and feet. Abnormality was conservatively defined as participant-reported extinction of vibration >10 seconds before examiner-identified extinction at the stem. For all three of these modalities, binarized variables were reported for each participant indicating the presence of any focal abnormality.

Proprioception was assessed using manual movement of the distal phalanx 20-30 degrees up or down with forced-choice reporting of the movement direction. Summed number of items correct (out of 20) was reported for each individual. Mechanical detection threshold was assessed using Von Frey monofilaments (Baseline 12-1662; 5-piece set) and coded as the geometric mean of the smallest filament weight detected on the dorsal surface of each extremity.

Evaluation of stereognosis was based on the protocol in Wingert 2008 [(12)](https://paperpile.com/c/ndoWsy/X0q54); participants identified five common objects (key, penny, pencil, spoon, and button) mounted to a smooth, wooden subsurface constraining haptic exploration. Each item was presented once to each hand in a pseudorandom order, and scores were reported as the number of correct responses out of ten.

Spatial tactile discrimination was evaluated using sets of acrylic Johnson/Van Boven/Philips (JVP) domes (Stoelting, Wood Dale, IL). An extended set of domes (12, 6.0, 3.0, 2.0, 1.5, 1.2, 1.0, 0.75, 0.5, and 0.35mm) was used given floor effects previously seen in individuals with CP [(13)](https://paperpile.com/c/ndoWsy/Ucc6), but testing and scoring protocols were otherwise unchanged. For each hand, after demonstration of each condition, a dome was pressed against the index fingertip for 1 second, and the individual identified whether ridges were aligned “up and down” or “side to side” (2-alternative forced-choice paradigm). Testing proceeded from the 12mm dome through successively finer ridge spacings until error rate exceeded 25% for a given ridge spacing. Each dome was applied 10 times in pseudo-random order of orientation (the same pseudo-random order was used for each participant, but a different order for each dome and each hand). The 25% error threshold for each hand was calculated via linear interpolation [(14)](https://paperpile.com/c/ndoWsy/IymOC); a threshold of 12mm was reported for individuals scoring >25% error at 12mm. The geometric mean of each hand’s thresholds is reported for each individual.

1. [Schiariti V, Fowler E, Brandenburg JE, Levey E, Mcintyre S, Sukal-Moulton T, Ramey SL, Rose J, Sienko S, Stashinko E, et al. A common data language for clinical research studies: the National Institute of Neurological Disorders and Stroke and American Academy for Cerebral Palsy and Developmental Medicine Cerebral Palsy Common Data Elements Version 1.0 recommendations. *Dev Med Child Neurol* (2018) **60**:976–986.](http://paperpile.com/b/ndoWsy/oWlLl)

2. [Palisano RJ, Rosenbaum P, Bartlett D, Livingston MH. Content validity of the expanded and revised Gross Motor Function Classification System. *Dev Med Child Neurol* (2008) **50**:744–750.](http://paperpile.com/b/ndoWsy/qaEep)

3. [Eliasson A-C, Krumlinde-Sundholm L, Rösblad B, Beckung E, Arner M, Ohrvall A-M, Rosenbaum P. The Manual Ability Classification System (MACS) for children with cerebral palsy: scale development and evidence of validity and reliability. *Dev Med Child Neurol* (2006) **48**:549–554.](http://paperpile.com/b/ndoWsy/8pzcW)

4. [Jethwa A, Mink J, Macarthur C, Knights S, Fehlings T, Fehlings D. Development of the Hypertonia Assessment Tool (HAT): a discriminative tool for hypertonia in children. *Dev Med Child Neurol* (2010) **52**:e83–7.](http://paperpile.com/b/ndoWsy/c4v7G)

5. [Bohannon RW, Smith MB. Interrater reliability of a modified Ashworth scale of muscle spasticity. *Phys Ther* (1987) **67**:206–207.](http://paperpile.com/b/ndoWsy/kdM0v)

6. [Barry MJ, VanSwearingen JM, Leland Albright A. Reliability and responsiveness of the Barry–Albright Dystonia Scale. *Developmental Medicine & Child Neurology* (1999) **41**:404–411. doi:](http://paperpile.com/b/ndoWsy/DBb8k)[10.1017/s0012162299000870](http://dx.doi.org/10.1017/s0012162299000870)

7. Rosenbaum, P., Paneth, N., Leviton, A., Goldstein, M., Bax, M., Damiano, D., ... & Jacobsson, B. (2007). A report: the definition and classification of cerebral palsy April 2006. Dev Med Child Neurol Suppl, 109(suppl 109), 8-14.

8. [Bastien CH, Vallières A, Morin CM. Validation of the Insomnia Severity Index as an outcome measure for insomnia research. *Sleep Med* (2001) **2**:297–307.](http://paperpile.com/b/ndoWsy/z1HL1)

9. [Kroenke K, Spitzer RL, Williams JBW, Löwe B. An ultra-brief screening scale for anxiety and depression: the PHQ-4. *Psychosomatics* (2009) **50**:613–621.](http://paperpile.com/b/ndoWsy/kmY9b)

10. [GrÉGoire J, Van Der Linden M. Effect of age on forward and backward digit spans. *Aging, Neuropsychology, and Cognition* (1997) **4**:140–149. doi:](http://paperpile.com/b/ndoWsy/afsFo)[10.1080/13825589708256642](http://dx.doi.org/10.1080/13825589708256642)

11. [Davis LE, Richardson SP. Neurologic Examination. *Fundamentals of Neurologic Disease* (2015)9–21. doi:](http://paperpile.com/b/ndoWsy/CrFHe)[10.1007/978-1-4939-2359-5_2](http://dx.doi.org/10.1007/978-1-4939-2359-5_2)

12. [Wingert JR, Burton H, Sinclair RJ, Brunstrom JE, Damiano DL. Tactile sensory abilities in cerebral palsy: deficits in roughness and object discrimination. *Dev Med Child Neurol* (2008) **50**:832–838.](http://paperpile.com/b/ndoWsy/X0q54)

13. [Sanger TD, Kukke SN. Abnormalities of tactile sensory function in children with dystonic and diplegic cerebral palsy. *J Child Neurol* (2007) **22**:289–293.](http://paperpile.com/b/ndoWsy/Ucc6)

14. [Johnson KO, Phillips JR. Tactile spatial resolution. I. Two-point discrimination, gap detection, grating resolution, and letter recognition. *J Neurophysiol* (1981) **46**:1177–1192.](http://paperpile.com/b/ndoWsy/IymOC)
